# Adapting in-person diabetes group visits to a virtual setting across federally qualified health centers

**DOI:** 10.3389/frhs.2022.961073

**Published:** 2022-11-11

**Authors:** Daisy Nuñez, Diana Marino-Nuñez, Erin M. Staab, Tracy Dinh, Mengqi Zhu, Wen Wan, Cynthia T. Schaefer, Amanda Campbell, Michael T. Quinn, Arshiya A. Baig

**Affiliations:** ^1^Department of Medicine, University of Chicago, Chicago, IL, United States; ^2^Pritzker School of Medicine, University of Chicago, Chicago, IL, United States; ^3^Midwest Clinicians' Network, East Lansing, MI, United States

**Keywords:** diabetes education, group visits, shared medical appointments, virtual, telehealth, health center, implementation, adaptation

## Abstract

Diabetes group visits (GVs) have been shown to improve glycemic control, enrich patient self-care, and decrease healthcare utilization among patients with type 2 diabetes mellitus (T2DM). While telehealth has become routine, virtual GVs remain understudied, especially in federally qualified health centers (FQHCs). We conducted a 5-year cluster randomized trial with a waitlist control group to test the impact of diabetes GVs on patients' outcomes in Midwestern FQHCs. Due to COVID-19, the 6 waitlisted FQHCs adapted to virtual GVs. FQHC staff were provided training and support to implement virtual GVs. The GV intervention included 6 monthly 1–1.5-h long education sessions and appointments with a primary care provider. We measured staff perspectives and satisfaction *via* GV session logs, monthly webinars, and staff surveys and interviews. Adaptations for implementation of virtual GV included: additional staff training, video conferencing platform use, decreased session length and group size, and adjusting study materials, activities, and provider appointments. Sites enrolled a total of 48 adults with T2DM for virtual GVs. Most FQHCs were urban and all FQHCs predominantly had patients on public insurance. Patients attended 2.1 ± 2.2 GVs across sites on average. Thirty-four patients (71%) attended one or more virtual GVs. The average GV lasted 79.4 min. Barriers to virtual GVs included patient technology issues and access, patient recruitment and enrollment, and limited staff availability. Virtual GV facilitators included providing tablets, internet access from the clinic, and technical support. Staff reported spending on average 4.9 h/week planning and implementing GVs (SD = 5.9). On average, 6 staff from each FQHC participated in GV training and 1.2 staff reported past GV experience. All staff had worked at least 1 year at their FQHC and most reported multiple years of experience caring for patients with T2DM. Staff-perceived virtual GV benefits included: empowered patients to manage their diabetes, provided patients with social support and frequent contact with providers, improved relationships with patients, increased team collaboration, and better patient engagement and care-coordination. Future studies and health centers can incorporate these findings to implement virtual diabetes GVs and promote accessible diabetes care.

## Introduction

Diabetes mellitus (DM) affects 30 million people in the U.S. (1). Type 2 DM (T2DM) accounts for 90–95% of cases of diabetes in adults (1). Adults with T2DM often face co-morbid chronic diseases (2, 3). The prevalence of diabetes is disproportionately higher among Hispanics (12.5%) and African-Americans (11.7%) compared to non-Hispanic whites (7.5%) (1). Hispanics and African-Americans have higher rates of diabetes-related complications, including amputations and CKD (4–6).

Federally qualified health centers (FQHCs) treat a larger proportion of patients with diabetes than other primary care physician offices (7). FQHCs also serve a high number of vulnerable patient populations, including patients of low socio-economic status (SES) and racial minorities (8), which have been disproportionately impacted by the pandemic. Research has shown that around 70% of patients in FQHCs have uncontrolled hemoglobin A1c values (9). Given this, FQHCs must optimize diabetes care to address population health needs.

The complex nature of diabetes care requires patients to sustain healthy lifestyle practices, manage their medications, and attend multiple provider visits. Diabetes group visits (GVs) provide an alternative form of diabetes care that consists of shared appointments with a diabetes educator in a group setting and an individual visit with a primary care provider (10). In this way, GVs add to the education and social support common to Diabetes Self-Management Education and Support (DSME) by incorporating a comprehensive medical visit to promote diabetes self-management. Diabetes GVs have been shown to effectively reduce hemoglobin A1c, improve self-management, and promote preventative care among patients (11–13). Despite the efficacy of diabetes GVs in improving patient outcomes and high staff satisfaction with GVs (13, 14), widespread integration of GVs into standard diabetes care in FQHCs remains limited.

The pandemic has required significant workflow modifications across FQHCs, such as increased telehealth visits to prevent the spread of this communicable disease (15). Telehealth visits play a critical role in the continuum of care for patients with multi-morbid chronic conditions, including diabetes (16). FQHCs utilized the opportunity to implement virtual diabetes GVs to adapt an effective care model to the trends of telehealth as well as increase the accessibility of diabetes care. Virtual GVs encountered barriers to implementation similar to individual telehealth visits, including technological access, resistance to change in clinical practice and cost challenges (17).

There is limited research that has systematically implemented and evaluated virtual GVs for adults with DM in the primary care setting. The aim of this research study was to adapt the diabetes GV research model to a virtual setting and to understand staff perspectives around the benefits, barriers, and facilitators to implementing virtual diabetes GVs across FQHCs.

## Methods

### Design

We conducted a cluster randomized trial with a waitlist control arm to test the impact of diabetes GVs on patients' outcomes in Midwestern FQHCs. The intervention framework is motivated by observed needs across four components in diabetes care: individual medical assessment, patient education, social support, and self-management. The University of Chicago research team partnered with the Midwest Clinicians' Network (MWCN), a non-profit corporation with membership consisting of FQHCs in ten Midwestern states, to conduct this trial. After an 18-month trial comparing GVs to usual care, FQHCs in the waitlist control arm received the intervention. Due to the COVID-19 pandemic, this intervention was modified to a virtual format. In this paper, we report only on the waitlist control arm's experience implementing virtual GVs. Results of the initial trial showed improved diabetes distress, social support, care knowledge, self-care, care self-efficacy, and quality of life among patients highly engaged in GVs and a text-messaging program across an in-person and virtual cohort. Further results from the initial trial will be reported separately.

### FQHC recruitment and training

FQHCs were recruited through the MWCN and filled out an application form to be included in the study. Applications were reviewed for FQHC characteristics, such as patient population, prevalence of T2DM among their patient panel, and form of patient insurance.

Sixteen FQHCs were randomized, 8 were assigned to the intervention and the remaining 8 were assigned to the waitlist control arm. Of the 8 FQHCs in the waitlist control arm, 3 withdrew, leaving 5 FQHCs in the control arm. FQHC 4 had two separate sites (sites 4a and 4b) participate in the study for a total of 6 sites. Each FQHC site needed to assemble an organizing team of three to four staff with at least one medical provider (e.g., physician, advanced practice nurse, or physician assistant). Originally, sites 4a and 4b had separate teams for in-person GVs, but for virtual GV implementation the same staff conducted GVs for both sites.

After 18 months, FQHCs in the waitlist control arm received training through a one-and-a-half day in-person training session in Chicago on how to conduct in-person group visits. At the session in early March 2020, staff from the University of Chicago and MWCN educated FQHC staff on GV structure and implementation, patient and staff recruitment, and potential barriers to GV implementation and success. However, prior to recruiting patients, due to the COVID-19 pandemic, the waitlist control arm from our trial had to quickly adapt to a virtual format. FQHC staff received 6 additional training webinars. There were 19 training and technical assistance webinars that lasted 1–1.5 h over the course of 15 months. We invited a clinical psychologist with experience leading virtual group therapy to present on effective utilization of telehealth services for groups. We also invited a pediatric endocrinologist and her research team to present on virtual type 1 diabetes group sessions (18–20). The research study team also reviewed research literature on benefits of virtual GVs, compiled tips for onboarding patients, created virtual GV planning worksheets, and shared ideas to inform staff training on implementing virtual GVs. FQHC staff were also trained on accessing REDCap, a secure web platform for building and managing online databases and surveys, to enter data and distribute surveys and enrollment forms. Most sites had a readily available telehealth platform which they were using for clinical visits, which they planned to use for the virtual group visits.

### Patient recruitment and enrollment

Upon consulting with experts in telehealth, our MWCN partners, and FQHC staff, it was decided that sites would enroll up to 12 patients for the virtual GVs, instead of up to 15 patients as we had done for in-person sessions, to facilitate virtual group discussion. Having a 12 patient limit was recommended by a licensed psychologist to promote social support in the virtual space and to accommodate for a shorter GV time of 1.5 h. Recruitment materials such as phone scripts and invitation letters were revised to inform patients that the GVs would be in a virtual format. As patients were being recruited, FQHC staff included additional questions such as what devices the patients would be joining from, if they had headphones, etc. to best help them set up for the virtual GVs. We recommended FQHC staff provide an orientation session with patients individually or as a group before the first GV session to introduce them to the video visit platform and to review the consent form and baseline survey. Consent forms, confidentiality agreements, and surveys were revised and converted to online formats. The consent forms were reviewed *via* phone or video with patients. Patients were given the options to complete forms in-person, over the phone, or returned *via* email or mail.

### Virtual group visit intervention

The FQHCs were asked to conduct 6 monthly 1–1.5 h long virtual GVs with up to 12 patients with uncontrolled T2DM (A1C ≥ 8%). Each visit was led by trained FQHC staff on a video conferencing platform. Additional guest speakers from various health professions provided group education at virtual GVs. Patients participated in facilitator-led group discussions that enabled material review and peer support. Patients were recommended to make a medical visit with a trained primary care provider within 2 weeks of each virtual GV.

To document the basic purposes that motivated the GV intervention, a Core Functions and Forms matrix (21) was used (Table 1). The motivating needs included access to comprehensive diabetes care, patient education, social support, and self-management. The core function column elaborates on the intended structural and procedural goal for each system need. Moreover, in the forms column we list the specific action items necessary to deliver each core function. The motivating needs, core functions and forms were all deduced by DN and DM and reviewed by AB. We also engaged in monthly webinars and conversations with FQHC staff to inform this adaptation framework. These core functions and forms were considered in the development of the virtual intervention.

**Table 1:** Function and forms model for in-person and virtual diabetes group visits.

**Motivating need**	**Core function**	**Forms**
1. Access to comprehensive diabetes medical care	1. Implement use of diabetes group visits and individual medical assessment in health center setting	• In-person or virtual learning sessions to train health center staff on implementation of group visit intervention
Need for improvement in quality of diabetes care *via* effective interventions		
		• Adapt to video conference call using a HIPAA-compliant telehealth platform for alternative access as necessary
2. Patient education	1. Improve patient knowledge about diabetes, nutrition, exercise, medication, and self-management	• Group education led by trained diabetes educator at appropriate health literacy levels
Limited understanding around diabetes disease process and care	2. Use of text messaging in diabetes care for diabetes education and self-management	• Use of CareMessage, a 25-week texting program that educates patients on diabetes, nutrition, exercise, stress management and medication
3. Social support	1. Create a space where patients with diabetes can connect and support each other in their care process	• Facilitate group conversations around diabetes care and coping skills
Need for support in the disease management		• Allow patients to have a family member or support person attend the group visit sessions with them if they choose
4. Self-management	1. Empower patients to take control of their diabetes, improve self-management, and make healthy lifestyle changes	• Identify needs and goals to help measure personal health progress
Facilitate care and goal setting		• Set aside individual goal setting sessions as needed

Motivating needs that influenced the development of diabetes virtual group visits intervention. Each motivating need has a core function depicting its purpose and the form in which each need was delivered in the intervention.

### Data collection and analysis

#### Session logs

Following each monthly virtual GV, FQHC teams completed session logs to record data about attendance, visit format, topics covered during visit, length of visit, presence of support people, patient location during visit, and additional education materials, services, or incentives provided to patients. Session logs also allowed for teams to reflect on what did or did not work well during the session. We used session logs to understand virtual group visit content and the ways in which the intervention was implemented at each FQHC.

#### Staff surveys

FQHC staff completed an enrollment team survey and a pre-training survey prior to the training session in Chicago measuring their attitudes about and confidence in implementing the GV model. As previously stated, the initial training session was in-person and following the onset of the COVID-19 pandemic, staff completed training for virtual GVs. All surveys after the initial training represent staff views on virtual GVs. They completed a post-survey after 6 months of virtual GVs evaluating the perceived impact of GVs on patients, clinicians, and the FQHC. Staff rated their agreement with survey items on a five-point Likert scale of “Strongly disagree,” “Disagree,” “Neither disagree or agree,” “Agree,” and “Strongly agree.”

#### Staff interviews

Post-intervention, trained research team members conducted 20–45-min telephone interviews with FQHC staff from June to September 2021. The interview questions were based on an interview guide designed to assess staff characteristics and involvement; barriers and facilitators to implementing and maintaining a virtual diabetes GVs intervention; characteristics of the virtual GV intervention as implemented and adapted to each site; desire and ability to sustain the GV intervention; and evaluation of the training. Interviews were audio recorded then transcribed by a professional transcription company for analysis.

#### Study documentation

Process data for the present study was retrieved from institutional review board (IRB) documents, progress reports, and training recordings. AURA IRB is an electronic research administrative system which facilitates research administration activities. To assess adaptations needed for research implementation of virtual GVs, we analyzed AURA IRB protocol amendments and any accompanying materials (e.g., surveys, confidentiality forms, consent forms, and planning worksheets). The IRB documents, surveys, training materials, and enrollment forms were updated by the co-authors and principal investigator to reflect necessary changes for virtual diabetes GV sessions. Study progress reports provided updates on project progress and project management for research funders. We also reviewed recorded training and technical assistance webinars to assess what additions the research team made to staff training for virtual diabetes GV implementation. To assess strategies FQHC staff incorporated to engage patients, we reviewed webinars and session logs where FQHCs reflected on their experiences with GV sessions. We also reviewed yearly continuing applications, where these experiences were summarized by co-author ES, and staff interviews where FQHC staff elaborated further on some of these experiences. We then compared the activities from the sample curriculum provided to the engagement strategies FQHCs shared to see what adaptations they made for virtual settings.

#### Statistical analysis

Descriptive statistics were assessed for survey data and linear mixed effect models were used to evaluate changes in attitudes before and after GV implementation.

#### Qualitative analysis of staff interviews

Four investigators used a modified template approach to text analysis using the interview guide to create an initial codebook (22). The transcripts were assigned to coder pairs using all possible combinations. Each member coded the assigned transcript independently then met with their partner to discuss to agreement. Further coding was done to identify subthemes and expand the codebook accordingly. NVivo 12 was used to code and organize the interview data.

## Results

### FQHC and staff characteristics

From the initial cluster randomized trial, 8 FQHCs with 9 clinic sites were assigned to the waitlist control group. One FQHC withdrew because they could not obtain institutional approval, another because of staff changes, and a third due to time and resource concerns. In the end, 5 FQHCs with 6 clinic sites were enrolled for implementation of virtual GVs. The 5 FQHCs were from Missouri, Illinois, Wisconsin, Indiana, and Iowa. Table 2 highlights FQHC characteristics including information about patient population and staff experience. Two FQHCs were urban, two suburban, and one rural. All FQHCs had previously held GVs for at least one health condition and at the time of enrollment, 83% (N=5/6) of FQHCs were having GVs for diabetes, heart disease, prenatal, or other conditions.

**Table 2:** FQHC characteristics, patient information, and staff experience.

**FQHC#**	**Setting**	**Number of patients**	**Public Insurance**	**Self-pay**	**Private Insurance**	**Number of staff on GV team**	**Staff with prior GV experience**	**Years caring for patients with T2DM mean (range)**	**Years working at FQHC site** ** mean (range)**	**Staff that attended training**
1	Urban	5,472	60%	17%	14%	4	2	25.3 (10–45)	6.3 (5–7)	3
2	Suburban	1,704	89%	5%	6%	5	0	13.5 (12–15)	1.3 (0.6–2)	5
3	Rural	11,605	56%	34%	10%	6	0	0.3 (0–1)	1.2 (0.2–1)	5
4	Urban	21,264	63%	13%	21%	11	1	8.2 (3–19)	3.2 (0.5–7)	10
5	Urban	17,389	63%	14%	22%	9	3	11.4 (2–20)	7.4 (0–19)	7

Data gathered from enrollment team surveys and staff pre-training surveys by FQHC.

There were 35 FQHC staff members enrolled throughout the 6 clinic sites. Twenty-two staff members attended the in-person training session in March 2020 and 30 staff attended at least one training and/or technical assistance webinar. All 5 FQHCs were represented by at least one staff member at all training sessions. Thirty-one (89%) completed the pre-training survey in February 2020. The mean age was 42.0 (SD = 11.1), 90% female, 61% non-Hispanic white, 16% African American, 16% more than one race, 3% Hispanic/Latino, and 3% Pacific Islander. The mean number of years in practice was 11.5 (SD = 9.0) and years providing diabetes care was 11.1 (SD = 10.8). One-third (*N* = 6/18) of staff had previous experience with GVs.

### Adaptations and implementation of virtual GVs

Table 1 denotes the adaptation model used for virtual GVs. Access to comprehensive diabetes medical care, patient education, social support and goal setting served as motivating factors for the interventions. Table 3 describes the adaptations made for the implementation of virtual group visits. There were adjustments to staff training, GV location, GV session time allotted, group size, patient recruitment and enrollment materials, survey administration, clinical measures, individual medical assessment, and education and interactive learning activities. All sites implemented virtual GVs. FQHC 2 held GVs from October 2020 to March 2021, FQHCs 4 and 5 from November 2022 to April 2021, FQHC 3 from December 2022 to May 2021, and FQHC 1 from March 2021 to August 2021. A total of 29 GVs were completed, and the average session duration was 82.1 (SD = 22.8) min. Seventeen of 35 (49%) staff members completed a post-GV survey 1 month after the 6th GV at their HC (from April to June 2021 and September 2021). Staff reported spending on average 4.9 h each week planning and implementing group visits (SD = 5.9). Majority, 65% (*N* = 11/17) of staff members were interested in continuing virtual GVs, and all were interested in participating in in-person groups. Staff attitudes toward GVs were compared from pre-training, when FQHCs were expecting to implement in-person GVs, to post-implementation of virtual GVs. Staff had improved awareness of barriers to GVs [3.8/5 (SD = 0.8) to 4.3/5 (SD = 0.5), *p* = 0.03] but were less confident in their FQHCs ability to sustain GVs [4.2/5 (SD = 0.6) to 3.7/5 (SD = 0.6), *p* = 0.01]. There was no significant change in staff's perception of the team's preparedness, motivation, or knowledge to implement or continue GVs. Measure of self-efficacy or awareness of what is needed to successfully implement GVs improved [3.3/5 (SD = 1.1) to 4.2/5 (SD = 0.5), *p* = 0.003].

**Table 3:** Adaptations for implementation of virtual group visits.

	**In-person**	**Virtual adaptations**
Staff training	In-person learning session with UChicago research staff in Chicago	· Learning sessions held *via* webinar
		· Additional training on virtual group visits (GV):
		o Explain benefits to virtual GVs
		o Share literature review of previous studies on virtual GVs
		o Host guest speakers to discuss facilitating virtual GVs
		o Consider mock virtual GV sessions
Location	· Private conference room, private clinic room, or other space available at the site	· Video conference call using a HIPAA-compliant telehealth platform
Time allocation	· Suggested time between 1.5 and 2 h	· Suggested time between 1 and 1.5 h to avoid teleconference fatigue
Patient recruitment	· Enroll up to 15 patients per group	· Enroll up to 12 patients per group
		· Revise recruitment phone scripts and letter invitations to reflect the virtual format of the intervention
		o Participating sites request patient email address to send REDCap forms
		o Assess patient capacity for virtual sessions (ask what device they will be joining from, if they have headphones, etc. to help them set up)
Confidentiality	·Patients sign confidentiality form at the first GV session	· Patients sign confidentiality form *via* REDCap and participating sites collect emails of any accompanying support person participating in GV sessions for the online REDCap form (emails are not accessible by study team)
Consent forms	·Staff/providers: Review the consent form and obtain written informed consent at the first learning session	· Staff/providers: Review the consent form *via* webinar then ask participants to print and sign the consent forms and return to the study team *via* mail.
	· Patients: Review consent form with patients before the first group visit session and obtain written consent from each intervention patient	· Patients: Contact patient (phone or video) to review consent, then email a personalized link to complete form *via* REDCap, or email, mail or pick up a copy of the consent form. The patient can return the signed consent form in person, by mail, or they can scan or take a photo of the signed consent form and email it to participating staff.
Surveys	· Staff surveys administered in-person after learning sessions	· Staff surveys administered online *via* REDCap
	· Patient surveys administered in-person prior to beginning the first group visit and after completing the sixth GV	
		· Patient surveys administered *via* email invitations to online REDCap surveys, verbally over the phone or *via* video call, mailed or emailed survey pdf version, or physical copy received and returned to participating sites by mail, scanned, or in-person.
		· Revise surveys to include virtual aspect and identify virtual-specific barriers and/or benefits to GVs
Clinical Measures	· Point of care testing	· In primary care visit
	· Patients check into the clinic for their GV appointment and have their vitals checked	· Drive up services for lab draws
	· Lab work if available at site	
Individual Medical Assessment	· Privately during group visits	· Recommended within 2 weeks before or after the group portion *via* phone, video, or in clinic as determined by each participating site
Education	· In-person activities such as cooking and physical activity demonstrations	· Activities adapted to virtual platforms
		· Use of innovative virtual games

Details of adaptations that took place for the implementation of virtual group visits. The left column lists the specific component that was altered, the middle column describes what procedures were done before the virtual implementation, and the last column outlines the adaptations for successful virtual group visit implementation.

From the 5 FQHCs recruited a total of 251 patients were spoken to about the study and 91 agreed to participate. Out of 160 patients who did not agree to participate, 85 were unable to participate mostly due to other scheduled responsibilities and 7 due to having no access to internet or devices; 50 were not interested because they did not think they needed more diabetes education or they were already going to other diabetic groups or specialists; 11 for unknown reasons; 5 lost to follow-up; and 9 were ineligible due to not having a cell phone/texting, hemoglobin A1Cs below 8 or no diabetes, and for being out of town. Of the 91 that agreed to participate, 42 were not enrolled mostly due to loss to follow-up, for being unable to participate, or were ineligible. In the end, a total of 49 patients were enrolled in the study. One patient was withdrawn prior to the first GV and is not included in the analyses.

Sites enrolled a total of 48 adults with T2DM for the virtual GVs, with baseline hemoglobin A1C 9.8 ± 1.8%, mean age 55 ± 12, 67% female, 67% African American, 27% non-Hispanic white, and 6.2% Hispanic. Table 4 encompasses information about GV eligibility, enrollment, and attendance by FQHC site. All FQHCs implemented GVs. Attendance ranged from 0 to 9 patients at GV sessions, and an average of 4 (3.8) patients attended each session across all FQHCs. Each patient attended a mean of 2.1 ± 2.2 GV sessions across sites. Thirty-four patients (71%) completed one or more virtual GVs and 14 patients attended no virtual GVs. Of the 34 patients that attended, 20 (59%) attended with video from home, 4 (11%) with phone only from home, 3 (9%) with video from clinic room, 3 (9%) with video from home and other/unknown location, 2 (6%) with video from home and clinic room, and 2 (6%) with video and phone only from home. For patient surveys at baseline 38 were completed and at 6 months 22 were completed for a total of 60. Of the 60 patient surveys, 42 (70%) were completed or returned in person, 6 (10%) by phone, 1 (2%) by mail, and 11 (18%) were unspecified. Those that were unspecified were reported as either majority being paper copies or mostly over the phone.

**Table 4:** Group visit (GV) eligibility, enrollment, and attendance (N) per site.

	**Eligible**	**Enrolled**	**GV1 N**	**GV2 N**	**GV3 N**	**GV4 N**	**GV5 N**	**GV6 N**	**Average GV duration (min)**	**Time staff spent planning and implementing GV (hours/week)**
Site 1	53	12	4	3	1	3	0	2	118.0	*M* = 5.5, SD = 3.54
Site 2	35	6	5	5	3	2	4	3	90.0	*M* = 1.17, SD = 0.76
Site 3	19	5	3	3	2	3	3	3	75.8	^*^
Site 4a	137	7	4	2	1	0	0	0	60.0	*M* = 13.0, SD = 9.89^**^
Site 4b	278	6	1	2	2	0	0	0	60.0	
Site 5	308	12	7	9	7	6	5	6	72.5	*M* = 3.6, SD = 4.72

Information about eligibility, enrollment, and attendance per site as reported in GV session logs. Data on time spent planning and implementing GVs was obtained from staff post-survey.

* Information not reported by staff.

** Site 4a and Site 4b shared the same staff.

### Barriers to implementing virtual GVs

The COVID-19 pandemic presented barriers to virtual GV implementation. As a result of COVID-19, FQHC staff had modified work environments (e.g., spacing, remote work), additional clinical tasks (e.g., administering vaccines) and less availability. FQHC 1 delayed GV implementation by about 4 months due to substantial staff turnover. In the post-GV staff survey, most staff cited other COVID-19 related priorities at the FQHCs as the biggest barriers for implementation. Additionally, during webinar check-ins, FQHC staff reported some patients felt restricted and isolated because of the pandemic.

Other patient-related barriers to implementation included recruitment and retention, patient attendance, internet and device access, and technology navigation. Common reasons for patients not participating were mostly due to scheduling conflicts or not being interested. Even after enrollment, some patients did not attend GVs (Table 5). Some patients did not have access to internet or devices. FQHC location in a rural area was an additional challenge for internet access and connectivity. Some patients also had difficulty navigating and logging into the video conference platform (e.g., patients continuously forgetting login credentials).

**Table 5:** Perceived challenges and benefits of virtual group visits among staff, *N* = 19.

**Theme**	**Subtheme**	**Selected staff quotes**
Challenges of virtual group visits	a. Technology	“the biggest challenge would probably be just the access of technology to our patients … making sure that everybody who was wanting to be involved had internet access, that they had something to join with, whether it was a phone or a tablet, computer.”
	b. Patient attendance	“the challenges of the virtual were getting the participants in to actually educate them on how to do the login … me not being a technical person myself. I had to get that education as well.”
	c. Adaptation to virtual contact	“Eventually we started to get patients that would confirm and not show up during the meeting. It went from tons of participation, everyone being excited to people were just too busy to come or they would again, register and all the information would be verified but then at the time of the Zoom meeting, nobody comes in.”
		“interpersonal benefit that's lost a little bit with that. But we were able to get a group that had a pretty good rapport and was quite engaged throughout. So that, was good.
		“cadence and the timing and how to make sure that no one person was monopolizing the conversation or speaking too much and how to make sure that one of the patients who was on her cell phone quite a bit, how to manage that. So it wasn't distracting to anyone else, how to get the quiet ones to speak. “
Health center recommendations for recruitment and retention	a. Provider recommendation	“our letters came from the physicians and then during their provider visits they were referring direct referrals over, and that was very helpful. Patients have a good relationship with their provider and there's a lot of trust there…the patients took that pretty seriously. So we thought that really helped with recruitment.”
	b. Honest description of expectations	“definitely explaining the program thoroughly when you were recruiting people [...] letting them also know that it's optional. Because I think sometimes people feel pressure to be in it, and they don't necessarily have the time. So definitely, starting off from the base to make sure that you have people that know what they're in, what they're expected of for the group, and then what's expected of us too. And then, just making sure that they can make that time commitment.”
	c. Provide incentives	“I do think incentives helped the retention of keeping those people that started. I think it helped keep them coming back each month to see, kind of what they'd learn and then what they might receive in the mail for participating.”
	d. Building rapport	“For retention, we had a lot of hands-on, we do each month kind of connecting with the persons each month to make sure that they had what they needed. So, I think that's kind of essential for keeping people going, even if it's even just once a month.”
		“I'd already built up a rapport with some of the patients that I had called and reached out to. So they kind of knew me already”
Benefits of virtual group visits	a. Health center	“I think for our organization, that's a benefit because we know tele-health has a benefit to our patients and if it's going to be an effective program in our system, then we need to, as the system, we need to be comfortable with it and sell it as a positive thing to our patients too.”
	b. Patients	“they would have the opportunity to speak with others that were going through some of the same things that they were going through … to be able to share how they overcame or how they were working through or dealing with some of their issues with diabetes”
		“it was nice to see was the support and the morale with the group. We had patients that were sharing their contact information with each other and were showing all the different ways that they have”
	c. Staff	“they just came together so well as a team and support each other and shared information and work together to provide good information to the patients. It was just wonderful to watch. I was just so excited and so happy about it.”

Selected quotes from post-group visit implementation staff interviews. The three themes and their respective subthemes and quotes below were selected for their relevancy to this manuscript. They reveal staff perspectives on barriers and facilitators for implementing diabetes virtual group visits.

Staff also experienced difficulties adjusting to technology, allotted time, and to virtual contact. As noted in Table 5, staff needed additional technical support. During interviews, staff mentioned adjusting to the virtual format during cooking and physical activity demonstrations was more challenging because of camera and sound manipulation (Table 6). From webinars, FQHC staff reported that it was difficult getting patients engaged with the time allotted and amount of material to cover.

**Table 6:** Ways FQHCs Engaged Patients in Virtual GVs.

**Topic**	**Virtual activities examples**
Physical Activity	*Cardio drum session*
	Purpose: demonstrate accessible physical activity (chair and low impact) to motivate patients to think outside the box for exercise
	Adaptations: use breakout rooms on telehealth platforms to have patients work in smaller groups or in partners after exercise demonstrations to encourage them to attempt the routines
	Challenges: patients and staff need their own exercise equipment; need to adjust camera positioning
	Suggestions: provide the necessary exercise equipment (exercise balls, sticks, and buckets) and deliver them well ahead of time; have one or several staff members use a handheld device while streaming on the telehealth platform to show different angles
Nutrition	*Recipe presentation*
	Purpose: learn about nutritional value of foods to encourage healthier food choices
	Adaptations: may supplement or replace a traditional cooking demonstration; have a volunteer prepare a recipe from the American Diabetes Associated Food Hut website or another reliable source and present its nutritional value (e.g., carbs, serving size, calories, taste, etc.); offer a grocery store gift card as an incentive; have patients recreate recipe at home so they can taste it as well
	Challenges: not everyone may have necessary ingredients available; allergies and dietary restrictions
	Suggestions: find a recipe with common ingredients and provide a list of substitutes well in advance; if within budget, deliver ingredients to patients; have a nutritionist or a registered dietician guest speaker present; plan for a mix of cultural foods
Education	*Emoji game*
	Purpose: identify and brainstorm how to treat symptoms of hyperglycemia and hypoglycemia
	Adaptations: designate an emoji for each symptom (ex. water drops for extreme thirst) then show each emoji on the screen and ask patients how they would treat the symptom the emoji represents
	Challenges: emojis differ across devices
	Suggestions: instead of sending the emoji through chat, share images of emojis on the screen so everyone sees the same emoji; use basic emojis available in most devices
Incentives	*Healthy gift basket, grocery store gift card, cookbook, tablets, coloring books, diabetic socks, self-care kits, kitchen supplies, portion plates*
	Purpose: to help maintain patients engaged and make them feel supported
	Adaptations: incentives may be delivered *via* mail for physical items or email for gift cards or other e-resources accessible through links, staff may also coordinate a time for patients to pick up from clinic
	Challenges: health centers may not have the funds to sponsor incentives
	Suggestions: pitch idea to stakeholders; apply for grants; find free resources for patients like activities to de-stress; motivational songs; hint at incentives when sending invitations; coordinate incentives to match learning topic

Description of activity, virtual adaptation, challenges, and suggestions for interactive learning strategies staff utilized to maintain patient engagement during the virtual group visit intervention.

Additional barriers to virtual GV implementation included reimbursement and incorporating the provider visit into sessions. FQHCs expressed they were not billing for the diabetes education portion of the virtual GVs. During webinars, staff expressed interest in learning more about billing, referrals, and insurance coverage. Other barriers included the provider not being present during all sessions and patient confusion about the team's provider role. Some FQHCs also reported experiencing difficulty incorporating provider visits with the GV session.

### Facilitators to implementing virtual GVs

The FQHCs developed various strategies for overcoming patient barriers to participation. Virtual GV facilitators included inviting patients who did not have devices or internet access at home to go to the FQHC and join virtually from individual clinic rooms. Access to Wi-Fi or internet connection was provided in 38% (*N* = 11/29) of virtual GVs. Some FQHCs also provided transportation for those patients who needed to go to the clinic site for internet access in 21% (*N* = 6/29) of sessions. Other facilitators included providing devices for patients (e.g., tablets, hotspots); allowing patients to call in without video if necessary; and mailing copies of materials ahead of time or having patients pick them up from the clinic. In 48% (*N* = 14/29) of the virtual GVs, patients were provided a tablet or device to participate in session. Patients also received incentives (e.g., gift cards, gift baskets, fresh produce delivery) and educational materials for 54 (*N* = 15/29) and 86% (*N* = 25/29) of sessions, respectively.

Some FQHCs provided a pre-session for technical support and training for both patients and staff prior to the first group visit; make-up sessions; 10-min breakout room sessions to get to know providers; and a 30-min “open house” before official GV start time to revisit guidelines, play games to review previous lessons and provide additional technological assistance. As noted in Table 6, the FQHCs thought of many creative ways to keep virtual sessions engaging and interactive, such as playing a game using emojis to identify symptoms of hyperglycemia and hypoglycemia and leading accessible physical activities like chair cardio drumming.

During post-intervention staff interviews, staff suggested recommendations that could improve recruitment and retention (Table 5). They suggested having providers recommend the program to patients, giving detailed descriptions of the virtual GV intervention, providing incentives, and building rapport with patients for better outcomes.

### Staff perceived benefits of virtual GVs

**Figure 1:**
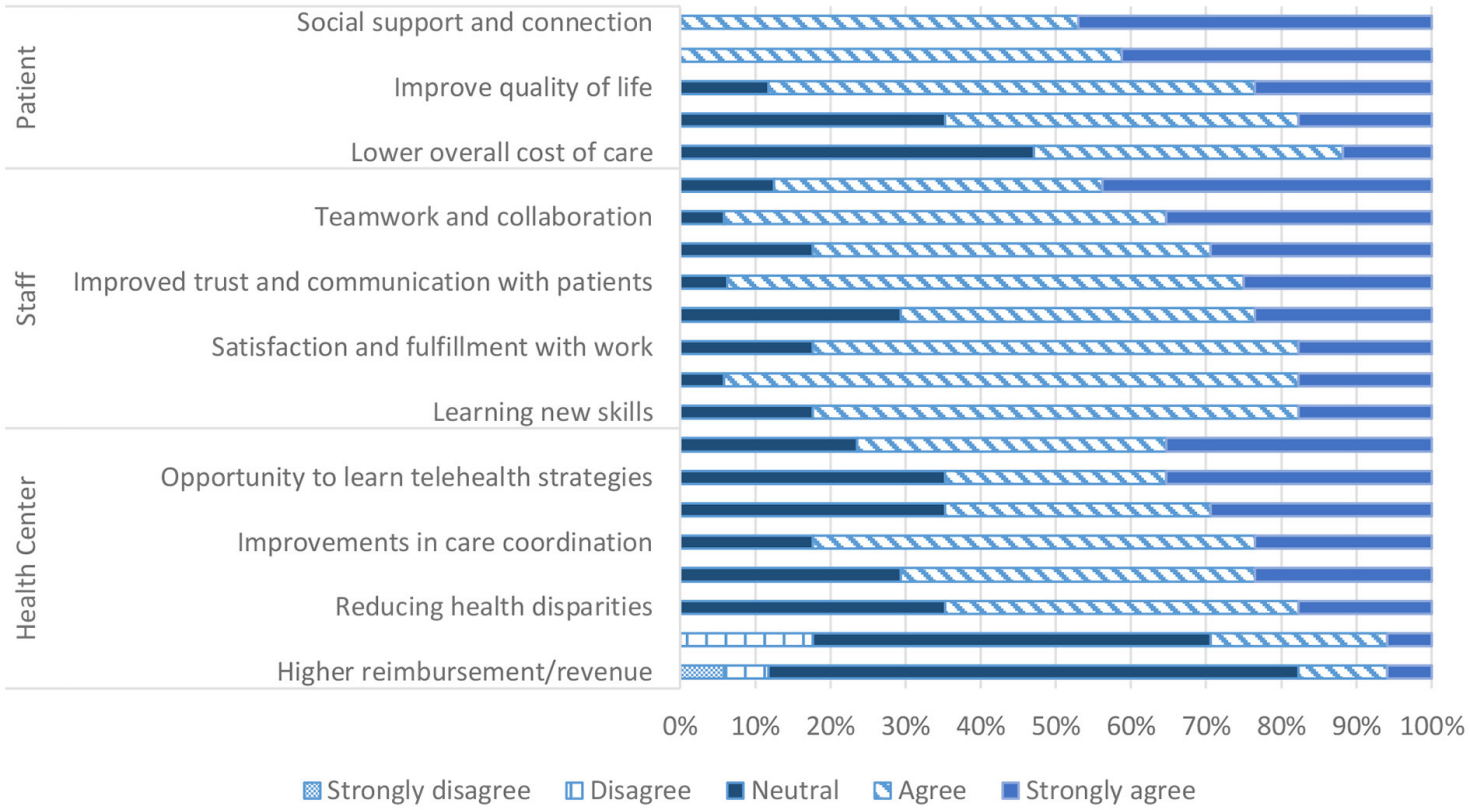
Staff perceived virtual GVs for patients, staff, and health center. Staff perceived virtual group visit benefits across three categories: patient, staff, and health center.

Figure 1 highlights staff perceptions of virtual GV benefits at the patient, staff, and FQHC level based on staff surveys.

#### Patient benefits

In terms of benefits of virtual GVs for the patient, all staff agreed that they empowered patients to manage their diabetes and provided patients with social support, connection, and more frequent contact with medical providers. Staff were least confident in the ability of virtual GVs to improve clinical outcomes and lower cost of care for patients with only 65 (*N* = 11/17) and 53% (*N* = 9/17) respondents agreeing that they do so respectively.

#### Staff benefits

Staff largely agreed with all proposed benefits to providers and staff. These included improved communication, trust, and understanding with patients, increased opportunity for teamwork, collaboration, and creativity, and more variety in their work. The least agreed upon statement was that virtual GVs allowed providers and staff to get to know each other with 71% (*N* = 12/17) agreeing.

#### FQHC benefits

There was greater variety in perceived benefits to the FQHC. Most staff agreed that virtual GVs lead to better patient engagement and care coordination as well as higher patient satisfaction. However, staff were less confident that virtual GVs increased provider productivity or led to higher reimbursements with only 29 (*N* = 5/17) and 18% (*N* = 3/17) staff members agreeing respectively.

## Discussion

Given the unpredictability of the COVID-19 pandemic, we modified the approach from in-person diabetes group visits to a virtual format across Midwestern FQHCs. Virtual GVs were implemented in all FQHC sites and staff found them beneficial. While the intervention's inclusion criteria and core components remained the same, additional consideration was needed for staff training, group size, recruitment and enrollment forms, and survey administration. Main challenges included technological barriers for both patients and staff, and patient recruitment and retention. Facilitators for virtual GVs included providing patients with tablets, orienting patients to the virtual platform, and incorporating creative activities for patient engagement. Successful outcomes included representation of all 5 FQHCs at training sessions and majority of staff interest in continuing virtual GVs.

All FQHCs implemented virtual GVs and staff found the intervention beneficial for patients, staff and the health center. Other studies on virtual visits or telehealth reported staff-perceived or patient-reported benefits such as improved self-efficacy (23) and peer support (23, 24) as general GV benefits. In addition, virtual specific GV benefits included time saving (24), scheduling and location flexibility (25, 26), and ease of participation due to reduced transportation barriers (25, 26). Our study is in agreement with these findings and adds additional perceived benefits. In our study, the most common staff-perceived benefits for patients included self-empowerment, improved quality of life, social support and connection. Staff felt virtual diabetes GVs improved trust and communication with patients, teamwork and collaboration, and better understanding with patients. While staff showed significant improvement in awareness of barriers and of what is needed to successfully implement GVs, as previously mentioned in the results, their confidence in their ability to sustain/implement GVs decreased. A possible explanation for this finding is their increased knowledge and awareness of challenges and barriers in virtual GVs led them to feel less confident about their ability to sustain the intervention. Specifically, the continuous outreach from staff in contacting patients and providing additional facilitators (e.g., devices, internet access, transportation, etc.) to improve retention yet having low attendance may have discouraged some staff members. Additionally, it is important to note that FQHC staff were expecting in-person GVs at the time of enrollment. Although 65% of staff were interested in continuing virtual GVs, all FQHC staff remained interested in participating in in-person GVs. A strong preference for in-person GVs and low acceptability of virtual GVs may lead to variation in sustainability confidence (27, 28).

Nonetheless, majority of staff agreed that virtual GVs benefited the FQHC's improvements in care coordination and offered an opportunity to implement an alternative model of care. Other studies report less staffing and overhead costs as additional network benefits (26). However, only a few staff in our study agreed with higher reimbursement/revenue as a perceived benefit for the FQHC. This may be because FQHCs billed for individual provider visits alone, but did not account for diabetes education. Overall, implementation and reported benefits of our intervention and that of other studies suggest virtual GVs are feasible and beneficial for patients, staff, and FQHCs across different health conditions. Our adaptation model is not limited to diabetes and may be of use to other health education programs interested in implementing virtual GVs.

Programmatic changes had to be made to adapt in-person diabetes GVs to a virtual format. First, staff training was modified to include education on virtual program implementation, barriers, and facilitators. Second, group size was modified to facilitate group interaction in a virtual setting and reduce risk of “Zoom fatigue” (29). Moreover, staff supported patient participation in virtual group visits to ensure evenly distributed conversation and engagement across patients. Third, enrollment forms and survey administration were made more accessible by providing various options for completion and return (e.g., by mail, email, over the phone, etc.). Of those that responded, majority returned the surveys in person or completed over the phone. No participants completed surveys electronically. As noted in staff interview results, this may be because existing rapport and repeated contact between FQHC and patients may encourage more engaged research participation. Future programs implementing virtual GVs may offer options by mail, email, over the phone, etc. to optimize patient response and later assess which format works best for them.

While adaptation to virtual GVs was accomplished, it was not without challenges. Other studies on transitioning to telehealth reported internet connectivity (25), access to technology (30), and participant login issues (25) as challenges. Interview and survey results from this study found similar challenges including technology access, technical concerns, and adaptation to virtual contact. Although internet and technology access remain an issue especially among minorities (31), virtual GV implementation sites may reduce these barriers by providing devices, Wi-Fi, and pre-sessions for technical support as the FQHCs in our study did. It is important to mention that the implementation of this study occurred earlier in the pandemic when not all FQHCs had telehealth platforms set up. This may explain why FQHC staff reported some difficulty getting accustomed to interacting with patients virtually. Considering telehealth services are now more widespread (32), situating patients and staff to telehealth may present a lesser challenge thereby making implementation more feasible. Nevertheless, FQHC staff were able to build rapport and maintain patients engaged despite these barriers.

When orienting patients with technology for virtual GVs, staff need to be comfortable navigating it as well. Other studies reported retraining staff and patients (30) and limited staff experience with software (25) as additional technology related challenges. As previously noted in barriers to implementation, some FQHC staff did not feel confident and needed additional technical support. While there was additional training on virtual group facilitation, telehealth services, and REDCap usage, there was no specific training on a given virtual platform (i.e., Zoom or Microsoft Teams). Instead, each FQHC used their own preferred virtual platform. This was done purposefully so FQHCs could use what was already available to them to facilitate rapid virtual GV implementation. With the rise of virtual care, telehealth is now more centralized with additional training and technology implemented to accommodate the shift (32). Even so, staff experience levels with technology should be assessed to provide additional technology support as necessary.

Another challenge FQHC staff faced was patient recruitment and retention. Challenges to patient recruitment and retention are seen across various lifestyle modification programs (33–35). In our study, additional challenges included the COVID-19 pandemic and the rapid transition to a virtual format. Even though poor patient recruitment and retention is common, building rapport and trust with patients, getting providers to recommend virtual GVs, providing incentives, and describing challenges and benefits of virtual GVs as FQHC staff did may help.

### Limitations

The present study has limitations that are important to consider in future application of this research. Given the rapid onset of the COVID-19 pandemic, the FQHCs in this study were asked to transition from in-person use of diabetes GVs to virtual ones. Clinical demands were higher with COVID-19 related services, therefore limited staff time to implement virtual GVs. Moreover, this rapid transition led FQHCs to implement a video platform that was familiar to them but was not consistent across sites. We recruited FQHCs from the Midwest Clinicians Network clinics, which while diverse, may not be generalizable across other regions and clinic networks.

### Future directions

Future programs seeking to implement virtual GVs should take into account various factors. FQHCs may need to budget for or apply for grants to fund any technological, software, or hardware support. Moreover, implementation timelines should incorporate time to address technological challenges and support for patients. Additionally, future programs may consider using a standardized virtual platform, ideally one that is familiar and with features that facilitate group discussion such as breakout rooms, screen sharing, chat boxes, and raise hand option. It is also important for staff to consider creative activities and modifications to timing and group size to lower risk of virtual fatigue. Holding a mock GV session or conducting all staff training on said platform may help orient staff to the virtual platform and address any challenges that may arise. Future programs may also consider providing staff with additional information on insurance coverage and billing and reimbursement for virtual GVs.

## Conclusion

In summary, FQHCs adapted diabetes GVs from in-person to a virtual platform during the COVID-19 pandemic. Modifications included changes in patient recruitment and enrollment, staff training, and learning to facilitate virtual sessions in a creative way to keep patients engaged. Challenges to implementation of virtual GVs included limited access to technologic support and lower staff availability due to pandemic demands. Facilitators of virtual GVs included providing technical assistance to patients, such as access to tablet devices, internet access from the clinic, technical support prior to GVs, and incorporating creative activities to engage patients in a virtual setting. Overall, FQHC staff reported overall satisfaction and support of future implementation of virtual GVs. Future studies should consider staff and patient support with technology and training modifications to facilitate the implementation of virtual diabetes GVs. Moreover, additional research should consider the ways to improve provider interaction with patients during GVs and include a control arm to assess the impact of virtual group visits on clinical outcomes.

## Data availability statement

The raw data supporting the conclusions of this article will be made available by the authors, without undue reservation.

## Ethics statement

The studies involving human participants were reviewed and approved by the University of Chicago Biological Sciences Division Institutional Review Board. The patients/participants provided their written informed consent to participate in this study.

## Author contributions

Study concept and design by AB, ES, DN, DM-N, MZ, WW, MQ, AC, and CS. Data acquisition was performed by DN and DM-N. Data analysis and interpretation were performed by AB, ES, DN, DM-N, MZ, WW, and TD. DN and DM-N wrote the initial manuscript draft. Critical revision of the manuscript for intellectual content was performed by all authors.

## Funding

This research was supported by the National Institute of Diabetes and Digestive and Kidney Diseases Chicago Center for Diabetes Translation Research (P30 DK092949) and the U.S. Department of Health and Human Services Office of Minority Health (1 CPIMP171145-01-00). Additional funding was received from the Dean's Office of the Biological Sciences Division of the University of Chicago. Study data were collected using REDCap, hosted by the University of Chicago Center for Research Informatics (NIH CTSA UL1 TR000430). AB was supported by a NIDDK Mentored Patient-Oriented Career Development Award (K23 DK087903-01A1).

## Conflict of interest

The authors declare that the research was conducted in the absence of any commercial or financial relationships that could be construed as a potential conflict of interest.

## Publisher's note

All claims expressed in this article are solely those of the authors and do not necessarily represent those of their affiliated organizations, or those of the publisher, the editors and the reviewers. Any product that may be evaluated in this article, or claim that may be made by its manufacturer, is not guaranteed or endorsed by the publisher.

## Author disclaimer

The contents of this manuscript are solely the responsibility of the authors and do not necessarily represent the official views of OMH.
